# Remineralization-Related Effects of Multi-Ion Cements on Demineralized Dentin

**DOI:** 10.1016/j.identj.2026.109753

**Published:** 2026-07-16

**Authors:** Zijuan Du, Tomoko Tabata, Masayuki Otsuki, Noriko Hiraishi, Chen Zeng, Yasunori Sumi, Yasushi Shimada

**Affiliations:** Department of Cariology and Operative Dentistry, Graduate School of Medical and Dental Sciences, Institute of Science Tokyo, Bunkyo-ku, Tokyo, Japan

**Keywords:** Deep caries, Dentin remineralization, Ion-releasing cements, S-PRG filler, Interim sealing

## Abstract

**Introduction and aims:**

This study investigates the short-term remineralization effects of multi-ion–releasing cements on demineralized dentin under simulated deep caries conditions and clarifies material-dependent differences relevant to interim caries management.

**Methods:**

Fifty root dentin specimens were assigned to five groups (*n* = 10): control (CO), Caredyne Restore (CR), Fuji IX (FU), HY Bond Temporary Cement Hard (HY), and IP Temp Cement (IP). Samples were demineralized (pH 4.5, 3 days), treated with cements, and subjected to 7-day pH cycling (4 hours/d at pH 4.5, 20 hours/d at pH 7.0). Mineral changes were assessed using swept-source optical coherence tomography before demineralization (DEM), after DEM, and after pH cycling. Transverse microradiography and scanning electron microscopy/energy-dispersive X-ray spectroscopy were used for quantitative mineral analysis and ion detection. Statistical analyses were conducted using one-way ANOVA and repeated measures ANOVA (*P* < .05).

**Results:**

All cement groups showed significantly reduced DEM compared to the control after pH cycling (*P* < .05). IP exhibited the most favourable remineralization-related response, as indicated by a reduction in attenuation coefficients towards baseline levels. Transverse microradiography analysis demonstrated significantly lower mineral loss and lesion depth in the CR, HY, and IP groups compared with the control. Scanning electron microscopy/energy-dispersive X-ray spectroscopy revealed material-dependent elemental signals, with zinc detected in CR and HY, fluoride-related elements in FU, and a distinct strontium signal in the surface prereacted glass ionomer–containing IP group.

**Conclusions:**

Ion-releasing cements demonstrated short-term protective effects and induced remineralization-related changes in demineralized dentin under the present in vitro pH-cycling conditions. Material-dependent differences were observed, with the surface prereacted glass ionomer filler–containing temporary cement showing the most favourable effects.

**Clinical relevance:**

Multi-ion–releasing temporary cements may contribute to the preservation of demineralized dentin and limitation of lesion progression when definitive restoration must be delayed.

## Introduction

Deep caries–affected dentin represents a structurally compromised substrate in which collagen exposure, mineral depletion, and enzymatic degradation may progress if immediate definitive restoration is not feasible.^1^^,^^2^ During staged or delayed caries management, the demineralized dentin should be stabilized to prevent further breakdown.^3^^,^^4^ Temporary cements provide short-term sealing; however, their ability to protect and remineralize deep caries–affected dentin remains insufficiently understood. Increasing evidence suggests that bioactive or multi-ion–releasing cements may support the survival of compromised dentin by enhancing mineral recovery and modulating the interfacial environment.^5^, ^6^, ^7^

Glass ionomer cements and polycarboxylate cements bond chemically to dentin through carboxylate–calcium interactions, offering reliable sealing and partial protection against microleakage-related irritation.^8^, ^9^, ^10^ Glass ionomer cements can release ions such as Ca^2+^, F^−^, and Sr^2+^, which contribute to acid buffering and suppression of demineralization (DEM).^11^ Surface prereacted glass ionomer (S-PRG) fillers further provide multivalent ion release, including F^−^, Sr²⁺, Na⁺, BO₃³^−^, Al³⁺, and silicate-related ions, together with acid-buffering activity, offering potential benefits for deep caries management.^12^, ^13^, ^14^ Caredyne Restore (CR), containing BioUnion bioactive glass, releases Zn^2+^, F^−^, and Ca^2+^, which may contribute to the preservation of demineralized dentin.^15^ While these materials align with the principles of minimally invasive dentistry,^16^^,^^17^ comparative evidence regarding their short-term remineralization effects on deep caries–affected dentin is scarce.

Swept-source optical coherence tomography (SS-OCT) enables nondestructive, depth-resolved monitoring of mineral changes.^18^, ^19^, ^20^ Transverse microradiography (TMR) is considered a laboratory gold standard for quantifying mineral loss and lesion depth.^21^ Using a laboratory model of deep caries–affected dentin, this study aimed to assess the short-term remineralization potential of two restorative and two temporary cements using a controlled pH cycling (PHC) model that simulates daily acid exposure. The null hypothesis was that there would be no significant differences in remineralization among the tested materials. A no-cement control group was included to clarify the protective benefits attributable to the materials.

## Materials and methods

### Specimen preparation

**Fig. 1:**
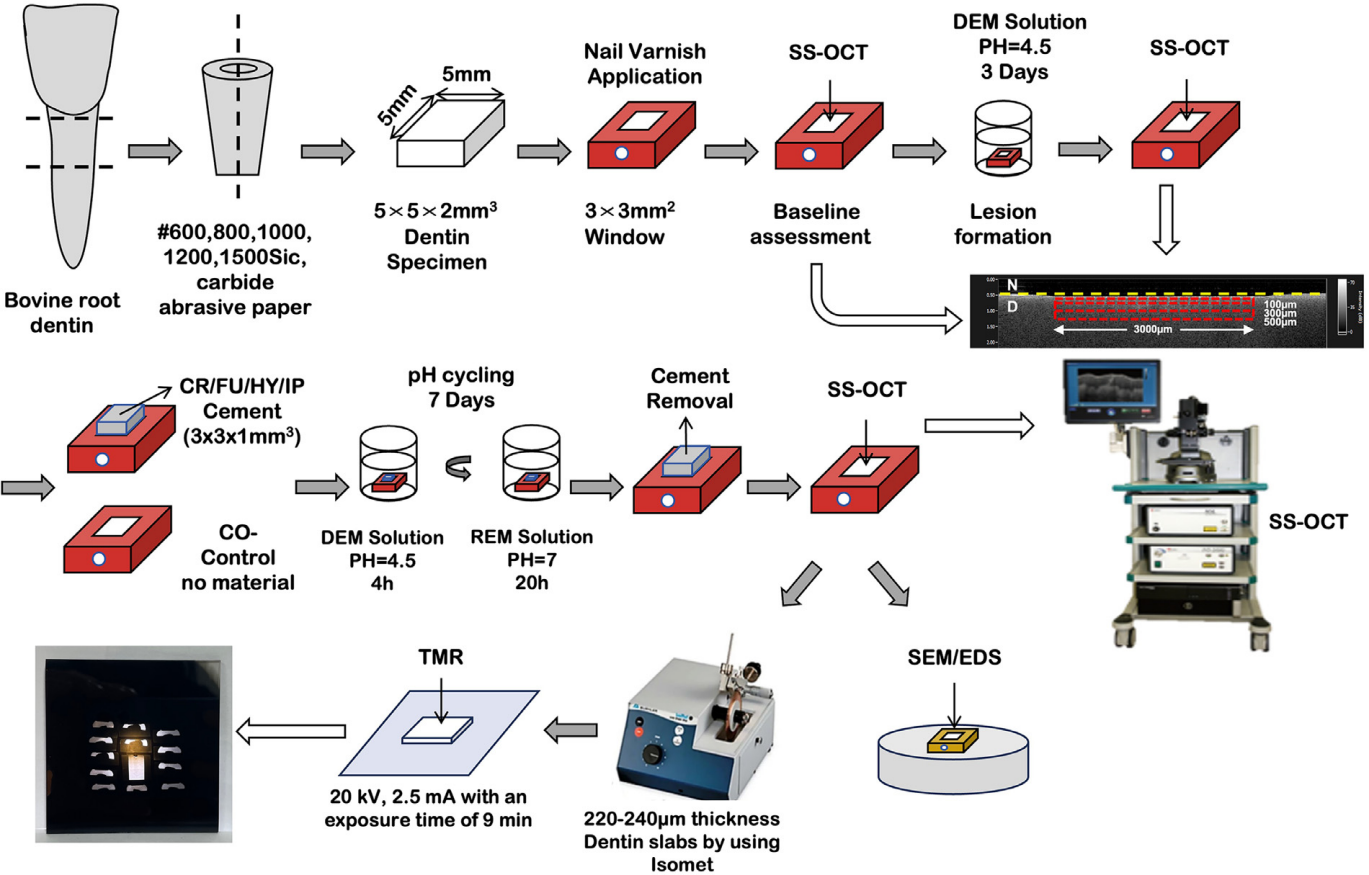
Schematic representation of specimen preparation. D, dentin; N, nail varnish. The yellow dashed line indicates the baseline, and the red dashed box denotes the region of interest (ROI; 3000 × 100, 300, and 500 µm²).

Freshly extracted, intact bovine incisor teeth were obtained as discarded biological materials through an approved procedure authorized by the Food Safety Commission of Japan, Ministry of Health, Labour, and Welfare. This study included 25 bovine incisors. After the removal of the crowns, residual soft tissues adherent to the root surfaces were carefully removed with a scalpel. Each root was then sectioned to obtain two standardized root dentin blocks measuring 5 × 5 × 2 mm³, resulting in a total of 50 specimens. The dentin blocks were embedded in a self-curing acrylic resin (UNIFAST II, GC). The exposed dentin surfaces were sequentially ground and polished under continuous water irrigation using silicon carbide abrasive papers with increasing grit sizes (600, 800, 1000, 1200, and 1500) to achieve flat and smooth surfaces. Subsequently, all specimens were coated with an acid-resistant nail varnish (Revlon), leaving a centrally defined window of 3 × 3 mm² to expose the dentin surface (Figure 1). The 50 specimens were randomly allocated to five experimental groups (*n* = 10) based on four different cements and one control: (1) cement-free control (demineralized dentin without cement application) – CO, (2) Caredyne Restore (GC) − CR, (3) Fuji IX (GC) – FU, (4) HY-Bond Temporary Cement Hard (Shofu) – HY, and (5) IP Temp Cement (Shofu) – IP. Table 1 provides detailed information on the materials used.

**Table 1:** Materials employed in this study.

Materials	Composition	Released ions	Company	Lot No.
Caredyne Restore (CR)	**Powder:** fluoroaluminosilicate glass, fluorozincsilicate glass (BioUnion filler), polyacrylic acid powder **Liquid:** polyacrylic acid, polybasic carboxylic acid, phosphoric acid, distilled water	Zn²⁺, Ca²⁺, F^−^, Si⁴⁺	GC Corp.	(P: 2403011, L: 2402201)
Fuji IX (FU)	**Powder:** fluoroaluminosilicate glass, polyacrylic acid powder **Liquid:** polyacrylic acid, polybasic carboxylic acid, distilled water	F^−^, Al³⁺, Si⁴⁺	GC Corp.	(P: 2311211, L: 2311241)
HY Bond Temporary Cement Hard (HY)	**Powder:** HY agent, zinc oxide, magnesia, silicon dioxide, pigment **Liquid:** poly (acrylic acid-tricarboxylic acid) sodium salt, water	Zn²⁺	Shofu Inc	(P: 072377, L: 022453)
IP Temp Cement (IP)	**Powder:** S-PRG filler, zinc oxide, magnesia, silicon dioxide **Liquid:** poly (acrylic acid-tricarboxylic acid) sodium salt, water, phosphoric acid	F^−^, Sr²⁺, Na⁺, Al³⁺, Si⁴⁺, BO₃³^−^	Shofu Inc	(P: 042456, L: 022421)

S-PRG, surface prereacted glass-ionomer.

BioUnion filler: bioactive glass particle composed of silicon dioxide (SiO2), zinc oxide (ZnO), calcium oxide (CaO), and fluorine. HY agent (tannin-fluoride): helps to minimize postoperative sensitivity and increase compressive strength. Released ions are based on material composition and previous studies.

### SS-OCT analysis

**Fig. 2:**
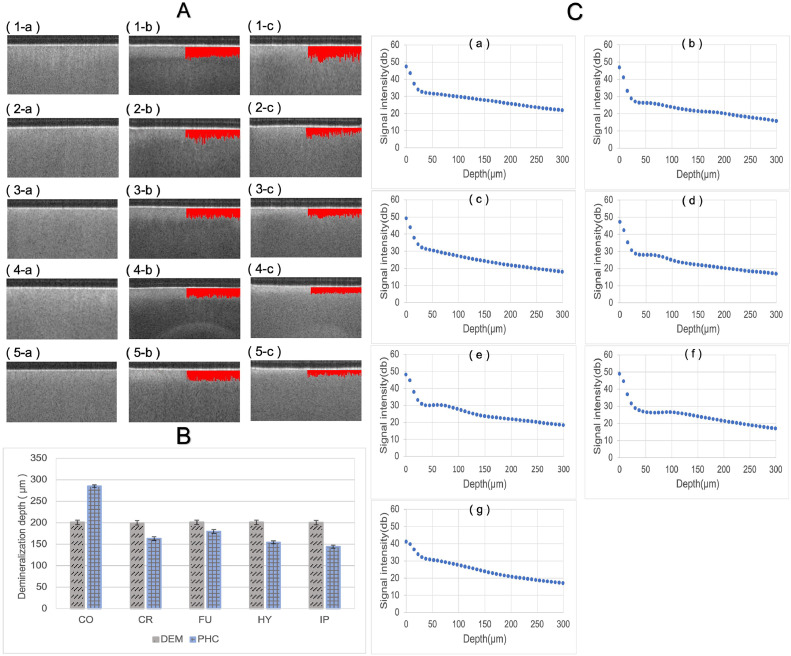
(A) Representative grayscale SS-OCT images (left) and the corresponding threshold-processed images (right) obtained from dentin specimens. Numbers 1 to 5 correspond to the CO, CR, FU, HY, and IP groups, respectively, and letters a to c indicate the three time points: before DEM (before demineralization), after DEM (after 3 days of demineralization), and PHC (after 7 days of pH cycling). (B) DEM depth (µm) for each group at DEM and PHC within a region of interest of 3000 × 500 µm². (C) Representative OCT signal profiles acquired before DEM (a, before demineralization), after DEM (b), and at PHC for the CO (c), CR (d), FU (e), HY (f), and IP (g) groups up to a depth of 300 µm.

SS-OCT (IV-2000; Santec) was conducted using a high-speed frequency-swept external cavity laser operating over a wavelength range of 1260 to 1360 nm at a sweep rate of 20 kHz.^22^^,^^23^ During scanning, the specimen’s surface was kept hydrated with deionized water to simulate in vivo conditions and minimize refractive index mismatches.^24^ Baseline OCT images were obtained before the DEM process, with the horizontal centre of each specimen selected as the observation site. The raw SS-OCT data acquired were analysed using ImageJ software (National Institutes of Health). To improve signal quality, a median filter was applied to suppress image noise. For each specimen, both DEM depth and attenuation coefficient (*µ_t_*) were calculated from the processed OCT signal profiles. Regions of interest (ROIs) extending from the dentin surface into the subsurface dentin were defined for quantitative analysis. A single ROI 3000 µm wide and 500 µm deep was used to determine DEM depth. For attenuation coefficient analysis, ROIs with the same width (3000 µm) and depths of 100, 300, and 500 µm were employed, with the superficial Fresnel reflection excluded from analysis (Figure 2B). Depth-dependent signal profiles were extracted using ImageJ, and the attenuation coefficient (*µ_t_*) was calculated based on an exponential decay model derived from the Beer–Lambert law.^20^^,^^25^^,^^26^

$$I\left(z\right)=ce^{-2\mu z}$$

where *I* represents the reflectivity signal intensity in (dB), c is a constant, and z is the depth from the surface, which has a factor of 2.

$$\mu_{t}\propto -\frac{1nI(Z)}{2z}$$

### Preparation of artificial root dentin lesions

Bovine root dentin specimens were subjected to artificial DEM by immersion in a solution containing 2.2 mmol/L CaCl₂, 2.2 mmol/L NaH₂PO₄, and 50 mmol/L acetic acid, adjusted to pH 4.5 with NaOH.^27^,^28^ The specimens were incubated at 37°C for 3 days to induce subsurface DEM with varying lesion depths and mineral loss values. For each experimental group, 10 specimens were individually immersed in 5 mL of the DEM solution, which was refreshed daily to ensure stable DEM conditions. After completion of the DEM period, all specimens were thoroughly rinsed with distilled water and examined using SS-OCT to confirm the formation of subsurface lesions. To enable reliable comparison, OCT scanning was performed in a consistent orientation for all specimens using premarked reference points.

### Treatment and pH cycling procedures

An artificial saliva solution was prepared, consisting of 1.5 mmol/L CaCl₂, 0.9 mmol/L KH₂PO₄, 130 mmol/L KCl, 1.0 mmol/L NaN₃, and 20 mmol/L HEPES, with the pH adjusted to 7.0 using KOH.^27^ Following the initial SS-OCT imaging conducted after 3 days of DEM, the exposed dentin window of each specimen was restored with the assigned cement according to group allocation. The cement was applied as a rectangular parallelepiped covering the 3 × 3 mm² surface window. Excess material was gently polished using 1500-grit silicon carbide paper under running water to achieve a uniform surface. The final thickness of the cement layer was approximately 1 mm. This thickness was selected to ensure complete coverage of the dentin window and to standardize the material volume for consistent ion release during PHC, rather than to simulate clinical occlusal conditions. Subsequently, all specimens were subjected to a 7-day PHC protocol to simulate dynamic oral conditions. Each specimen was alternately immersed in 5 mL of DEM solution (pH 4.5) for 4 hours and 5 mL of artificial saliva (pH 7.0) for 20 hours at 37°C in an incubator-shaker system (100 rpm). The solutions were refreshed daily to maintain consistent ionic activity. Upon completion of PHC, cement removal was gently performed after rinsing the specimens with distilled water, using an ultrasonic tip under controlled conditions.^28^ This procedure was conducted to allow direct evaluation of dentin surfaces without interference from the overlying cement layer. Care was taken to minimize alteration of the dentin surface during cement removal. The lesion formation was analysed by SS-OCT, maintaining the same orientation as the baseline scans by referencing premarked positions on each specimen. OCT imaging was performed both after 3 days of DEM and 7 days of PHC to evaluate changes in DEM depth and structure.

### TMR analysis

After SS-OCT observation, 25 randomly selected specimens (*n* = 5 per group) were assigned for TMR analysis. From these, 50 dentin slices (*n* = 10 per group) with a thickness of 220 to 240 µm were prepared using a low-speed diamond saw (Isomet; Buehler). To prevent dehydration and dimensional shrinkage, each section was stored in 70% glycerin before imaging. The specimens were then mounted on a high-resolution glass plate (Konica Minolta) alongside a 15-step aluminium reference wedge for mineral calibration. Microradiographs were acquired using an X-ray generator (SOFTEX CMR-2; Softex) operating at 20 kV and 2.5 mA, with an exposure time of 9 minutes. The developed radiographs were digitized using an optical microscope (BX41; Olympus) equipped with a CCD camera (DP70; Olympus). Quantitative analysis of mineral density profiles was conducted using ImageJ software (version 1.42q; National Institutes of Health) together with customized image-processing algorithms to determine integrated mineral loss (Δ*Z*, vol%·µm) and lesion depth (LD, µm).

### Scanning electron microscope (SEM) and energy-dispersive X-ray spectroscopy (EDS) analysis

Following SS-OCT observation, the remaining dentin specimens in each group (*n* = 5) were prepared for SEM and EDS. Specimens were fixed in 2.5% glutaraldehyde for 24 hours, rinsed with phosphate buffer, and dehydrated through a graded ethanol series (50%, 70%, 80%, 90%, 95%, and 100%). For SEM imaging, the specimens were air-dried overnight and sputter-coated with a thin platinum layer (E-102; Hitachi). SEM observations were conducted at 5.0 kV to evaluate surface microstructural changes in dentin.

For EDS analysis, the dehydrated specimens were coated with carbon to avoid masking low-energy elemental peaks. Elemental mapping was performed at 15.0 kV and 3000 × magnification to assess the surface distribution of calcium (Ca²⁺), fluorine (F^−^), zinc (Zn²⁺), aluminium (Al³⁺), silicon (Si⁴⁺), and strontium (Sr²⁺).

### Ion release analysis

Ion release analysis was performed to characterize the elemental release from the tested cements. Cement specimens were prepared by mixing the powder and liquid components according to the manufacturers’ instructions. Pellets (approximately 5 g each) were allowed to set at 37°C for 24 hours. The set specimens were then ground into powder and immersed in 50 mL of acetate buffer (pH 4.5) in polystyrene tubes, followed by incubation at 37°C under constant stirring for 24 hours. The suspensions were centrifuged, and the supernatants were collected and filtered. Elemental analysis was conducted using inductively coupled plasma, and fluoride ion concentration was measured using a fluoride-selective electrode.^29^ Detailed results are provided in Supplementary Table S1.

### Statistical analysis

Two statistical approaches were applied in this study. Changes across the three time points were analysed using repeated measures analysis of variance (ANOVA) with Bonferroni correction, while intergroup comparisons among the five experimental groups were evaluated using one-way ANOVA. Post hoc analyses were performed using either Tukey’s HSD or Bonferroni tests according to the assumption of variance homogeneity. All statistical analyses were performed using SPSS software (version 26.0; IBM). The sample size was determined based on comparable previous in vitro studies using similar dentin remineralization models and analytical methods. Statistical power analysis was additionally conducted using G*Power software (version 3.1) based on the primary SS-OCT and TMR outcomes after PHC, confirming that the sample size provided statistical power greater than 0.99. A significance level of *P* < .05 was adopted for all analyses. Normality and homogeneity of variance were confirmed using the Shapiro–Wilk test and Levene’s test prior to ANOVA.

## Results

### SS-OCT analysis

Figure 2A presents the representative original grey-scale SS-OCT images, together with the corresponding threshold-processed images that highlight high-scattering demineralized regions. After DEM, all groups exhibited a bright superficial zone, with the CO group demonstrating the greatest extent. In the threshold-processed images, this appeared as a large, red-marked lesion area. Following PHC, this area decreased in all cement groups – particularly in the S-PRG temporary cement (IP) – whereas the CO group showed a further increase in the lesion area.

Figure 2B and Table 2 present the quantified DEM depths. After the DEM phase, lesion depth increased significantly in all cement-treated groups compared to baseline values (*P* < .05), indicating successful lesion induction. Following PHC, these groups demonstrated significantly reduced depths relative to their DEM values (*P* < .05). Conversely, the CO group exhibited a further significant increase in DEM depth after PHC (*P* < .05), indicating continued mineral loss during the neutral phase. Among the cement groups, the IP group showed the smallest lesion depth after PHC, followed by HY, CR, and FU, reflecting their relative remineralization capability.

**Table 2:** Depth of demineralization and attenuation coefficient (μt) values of dentin at different experimental stages.

Depth of demineralization in 500 µm ROI depth for each group					
ROI: 3000 × 500 µm²	CO	CR	FU	HY	IP
Before DEM	0^A^	0^A^	0^A^	0^A^	0^A^
DEM	200.79 ± 7.09^B,a^	199.61 ± 11.15^B,a^	200.76 ± 4.46^B,a^	200.8 ± 3.41^B,a^	200.28 ± 6.35^B,a^
PHC	285.49 ± 2.81^C,a^	163.11 ± 4.3^C,b^	179.71 ± 4.74^C,c^	154.4 ± 3.26^C,d^	144.37 ± 3.36^C,e^
Mean ± SD (µm), *n* = 10					

Attenuation coefficient (*µ_t_*) of the three ROIs for each group					
	CO	CR	FU	HY	IP

**ROI: 3000 × 100 µm²**					
Before DEM	2.28 ± 0.29^A,a^	2.21 ± 0.32^A,a^	2.07 ± 0.27^A,a^	2.15 ± 0.18^A,a^	2.13 ± 0.21^A,a^
DEM	3.14 ± 0.5^B,a^	3.11 ± 0.2^B,a^	3.08 ± 0.28^B,a^	3.08 ± 0.27^B,a^	3.15 ± 0.17^B,a^
PHC	3.02 ± 0.41^B,a^	2.97 ± 0.17^B,ab^	2.76 ± 0.26^B,ab^	2.62 ± 0.3^C,b^	2.61 ± 0.25^C,b^
**ROI: 3000 × 300 µm²**					
Before DEM	0.86 ± 0.19^A,a^	0.96 ± 0.14^A,a^	0.91 ± 0.09^A,a^	0.97 ± 0.15^A,a^	0.99 ± 0.11^A,a^
DEM	1.34 ± 0.29^B,a^	1.23 ± 0.35^B,a^	1.23 ± 0.19^B,a^	1.31 ± 0.2^B,a^	1.3 ± 0.35^B,a^
PHC	1.38 ± 0.25^B,a^	0.91 ± 0.14^AB,b^	0.99 ± 0.06^C,b^	0.93 ± 0.03^A,b^	0.91 ± 0.05^A,b^
**ROI: 3000 × 500 µm²**					
Before DEM	0.77 ± 0.15^A,a^	0.75 ± 0.09^A,a^	0.74 ± 0.07^A,a^	0.77 ± 0.1^A,a^	0.81 ± 0.08^A,a^
DEM	1.23 ± 0.24^B,a^	1.15 ± 0.28^B,a^	1.11 ± 0.2^B,a^	1.21 ± 0.18^B,a^	1.28 ± 0.3^B,a^
PHC	1.16 ± 0.17^B,a^	0.88 ± 0.09^C,b^	0.93 ± 0.12^B,b^	0.93 ± 0.07^C,b^	0.88 ± 0.06^C,b^
Mean ± SD (mm^−^¹), *n* = 10					

Different uppercase letters indicate significant differences among time points within the same material, whereas different lowercase letters indicate significant differences among materials at the same time point (*P* < .05).

Signal intensity profiles of SS-OCT were analysed and are shown in Figure 2C. Signal intensity in the DEM group (b) declined sharply with depth, reflecting pronounced subsurface mineral loss. Cement groups after PHC (d-g) demonstrated intermediate profiles between those of before DEM (a) and the CO group after PHC (c), with high surface intensity that gradually decreased.

Table 2 presents the attenuation coefficient (*µ_t_*) values. Across all groups, *µ_t_* increased significantly after DEM (*P* < .05). Following PHC, no significant changes were observed in the CO group (*P* > .05), whereas the HY and IP groups exhibited significant reductions in *µ_t_* (*P* < .05) at all ROI depths, with material- and depth-dependent differences. FU showed a significant decrease at 300 µm, while CR demonstrated reductions at 500 µm. After PHC, *µ_t_* values in FU (100 and 500 µm) and CR (100 and 300 µm) approached their before-DEM levels. Across all depths, the CO group maintained significantly higher *µ_t_* values at 300 and 500 µm than the cement groups (*P* < .05).

### TMR analysis

**Fig. 3:**
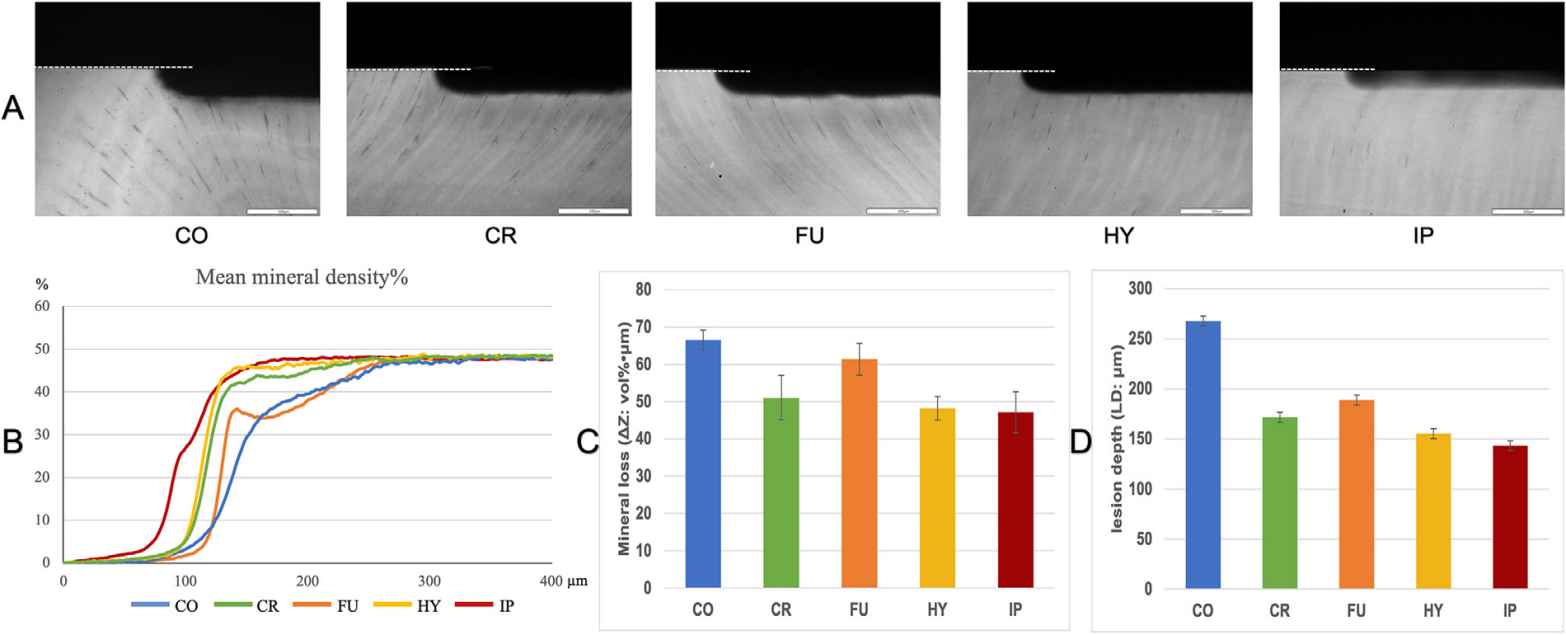
(A) TMR images illustrating mineral distribution after 7 days of PHC. The dentin surface is indicated by a white dashed line, and the shaded region represents mineral loss (Δ*Z*) and lesion depth (LD). (B) Mean mineral density profiles following PHC for the five experimental groups. The mineral density of the baseline reference region before demineralization was set at 48% (v/v) and used as the reference for normalization in ImageJ-based analysis. (C) Quantitative comparison of integrated mineral loss (Δ*Z*) among the experimental groups. (D) Quantitative comparison of lesion depth (LD) values among the experimental groups.

Table 3 and Figure 3 summarize mineral loss, lesion depth measurements, and representative mineral distribution profiles. No significant difference in mineral loss was observed between the control (CO) and Fuji IX (FU) groups (*P* > .05). Conversely, the CO group exhibited the greatest lesion depth, with values significantly greater than those of all cement-treated groups (*P* < .05). Among the materials evaluated, the IP group showed significantly reduced Δ*Z* and LD values compared to the CO group (*P* < .05).

**Table 3:** Mineral loss (ΔZ) and lesion depth (LD) of dentin after 7 days of pH cycling.

Mineral loss (Δ*Z*: vol%·μm) and lesion depth (LD: μm) of dentin after 7 d of pH cycling					
	CO	CR	FU	HY	IP
Δ*Z*	66.56 ± 2.65^a^	51.05 ± 5.96^b^	61.38 ± 4.28^a^	48.21 ± 3.19^b^	47.11 ± 5.48^b^
LD	267.5 ± 19.33^a^	171.5 ± 39.76^bc^	188.8 ± 32.13^b^	155.3 ± 23.25^bc^	143.2 ± 12.28^c^
Mean ± SD, n = 10					

Different lowercase letters indicate significant differences among materials (*P* < .05).

### SEM/EDS analysis

**Fig. 4:**
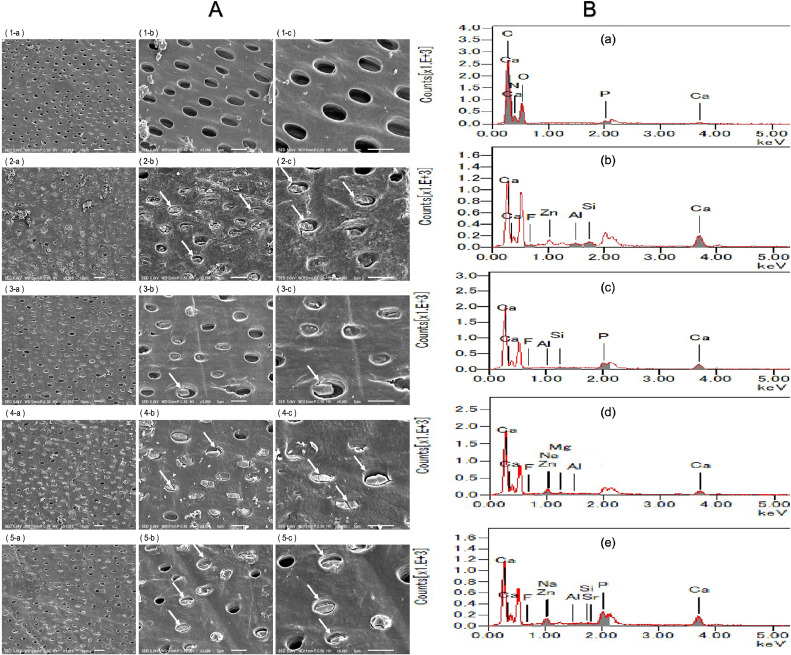
(A) Representative SEM images from 1 to 5 indicate CO, CR, FU, HY, and IP groups; the letters a, b, and c indicate the magnifications of 1000, 3000, and 5000 for SEM observation. White arrows indicate reactional deposits on subsurface dentinal tubules. (B) Representative EDS images: (a) CO group, (b) CR group, (c) FU group, (d) HY group, and (e) IP group.

Representative SEM images obtained after PHC are presented in Figure 4A. In the CO group, dentinal tubules remained open, and the peritubular dentin structure was clearly visible, indicating ongoing DEM without surface coverage. Conversely, all cement groups (CR, FU, HY, and IP) exhibited mineral-like surface deposits partially covering or occluding the dentinal tubules. FU showed a more granular surface morphology, whereas CR and HY presented denser precipitates adhering to the dentin surface. IP demonstrated the most uniform and continuous surface layer with consistent tubule coverage, consistent with its favourable remineralization-related changes observed in OCT and TMR analyses.

The corresponding EDS spectra are presented in Figure 4B. The CO group showed only Ca^2+^ and PO_4_^3−^ peaks originating from dentin. In the CR and HY groups, distinct Zn^2+^ peaks were detected, reflecting their zinc-containing compositions. FU exhibited pronounced F^−^, Si^4+^, and Al^3+^ peaks typical of conventional fluoroaluminosilicate glass ionomer. The IP group showed a distinct Sr²⁺ signal, along with F^−^, Si⁴⁺, Al³⁺, and Na⁺, consistent with the presence of S-PRG filler and its characteristic multi-ion release profile. Compared to the CO group, all cement groups displayed additional elemental signals, particularly F^−^ and Al^3+^, indicating ion exchange and deposition of material-derived ions on the dentin surface.

## Discussion

The present study evaluated the remineralization potential of various ion-releasing cements using a multimodal analytical approach. Therefore, the null hypothesis was rejected. Our findings demonstrated that all tested cement groups inhibited the progression of DEM and promoted mineral gain compared to the noncovered control group. The DEM protocol successfully produced caries-like subsurface lesions, and the subsequent PHC model confirmed that the application of these cements provided a protective effect against cariogenic challenges. SEM observations revealed mineral-like surface deposits partially covering dentinal tubules in all cement-treated groups, whereas the control group showed open tubules and exposed peritubular dentin, indicative of ongoing DEM. Central to this analysis was the use of SS-OCT, which revealed a marked increase in the attenuation coefficient (*µ_t_*) after initial DEM, reflecting enhanced light scattering due to structural disruption of dentin, as previously reported.^19^^,^^20^ Following PHC, significant reductions in *µ_t_* and lesion depth were observed in the cement-treated groups. Importantly, these optical findings were corroborated by TMR analysis, the gold standard for quantifying mineral loss (Δ*Z*) and lesion depth. Additionally, supportive morphological and elemental analyses using SEM/EDS provided complementary information regarding material-dependent surface changes observed after PHC. Together, these findings strengthen confidence in the observed material-dependent trends, as changes in OCT attenuation reflect alterations in the optical properties associated with structural and mineral changes in dentin.^25^^,^^26^

The PHC model used in this study was designed to simulate short-term acid challenges and neutral recovery phases that occur in the oral environment. These conditions may provide useful insights into staged or delayed caries management, where immediate, complete caries removal and definitive restoration are often avoided to reduce the risk of pulpal exposure.

Contemporary minimally invasive strategies, including selective caries removal and stepwise excavation, emphasize the preservation of caries-affected dentin while relying on effective sealing to arrest lesion progression and preserve affected dentin.^3^^,^^30^ Under these circumstances, interim sealing materials are required to not only provide mechanical coverage and bacterial isolation but also influence the physicochemical environment of the remaining dentin. Clinical and microbiological studies have demonstrated that sealing carious dentin without complete excavation can substantially reduce bacterial activity and slow or arrest lesion progression.^31^^,^^32^ Consistent with these observations, the present study showed that all cement-treated groups exhibited reduced lesion depth and mineral loss compared to the nonsealed control, supporting the concept that temporary materials may actively contribute to the protection of demineralized dentin and limitation of lesion progression rather than acting merely as passive barriers.^33^

Among the tested materials, the S-PRG filler–containing temporary cement (IP) exhibited the most favourable remineralization-related response. This superior behaviour was characterized by the greatest reductions in lesion depth, mineral loss (Δ*Z*), and attenuation coefficients (*µ_t_*), with *µ_t_* values at deeper ROIs approaching the pre-DEM baseline values. These findings are clinically relevant in situations where stable provisional sealing is required for extended periods before definitive restoration. The enhanced remineralization observed in the IP group is consistent with previous reports on S-PRG-based materials, characterized by their ability to release multiple bioactive ions, including fluoride (F^−^), strontium (Sr²⁺), and borate (BO₃³^−^).^12^^,^^14^^,^^15^ EDS analysis further confirmed the presence of material-derived elements on the dentin surface, demonstrating a distinct Sr²⁺ signal in the IP group. This finding suggests that S-PRG filler-derived ions may be associated with the favourable protective effects and mineral-related changes observed in the IP group. These ions may contribute to the observed protective effects through acid-buffering activity, improved resistance to mineral loss, and inhibition of further dentin DEM. Particularly, strontium has been suggested to partially substitute for calcium in apatite crystals, thereby promoting mineral nucleation, improving crystal stability, and increasing resistance to acidic dissolution, while fluoride contributes to the suppression of DEM and stabilization of mineral phases.^11^^,^^15^ The coexistence of fluoride, strontium, and other glass-derived ions therefore suggests a potential combined effect of multiple released ions that may be more effective than fluoride release alone in protecting demineralized dentin and limiting lesion progression. Additional ion release analysis was performed, and the full data are provided in Supplementary Table S1. The results demonstrated material-dependent ion release profiles and supported the interpretation that the observed protective effects and remineralization-related changes may be associated with the combined effects of multiple ions rather than a single dominant ion. Although EDS confirmed the presence of material-related ions on the dentin surface, the present study does not directly demonstrate their incorporation into the dentin structure or confirm synergistic remineralization mechanisms.

CR and HY also showed protective effects against further DEM, as reflected by their reduced lesion depth and mineral loss values. EDS analysis confirmed the presence of zinc-derived signals on the dentin surface, which may partly explain their protective behaviour. Zinc ions are known to inhibit dentin matrix metalloproteinases, thereby limiting collagen degradation within demineralized dentin and preserving the organic scaffold required for effective remineralization.^34^^,^^35^ Additionally, zinc may contribute to the formation of zinc-substituted calcium phosphate phases that facilitate mineral redeposition and enhance structural stability.^36^^,^^37^ These mechanisms are particularly relevant in deep dentin, where the preservation of collagen integrity is essential for lesion arrest.^38^

In contrast, FU exhibited comparatively limited protective effects despite its fluoride-releasing capability. While fluoride is effective in suppressing surface DEM, its ability to induce mineral recovery in deeper regions may be limited under short-term conditions, particularly in the absence of additional bioactive ions. The lack of zinc release in FU may partly account for its weaker performance at greater depths, suggesting that fluoride alone may be insufficient to provide the same level of protective effects in deeper regions of demineralized dentin during interim management periods.^11^^,^^13^

Collectively, these findings indicate that the qualitative composition of released ions may contribute to short-term protective effects on demineralized dentin. Although artificial DEM models cannot fully replicate the biological complexity of natural caries, the consistent agreement among SS-OCT, TMR, and SEM/EDS outcomes supports the validity of this model for comparative evaluation of ion-releasing materials. Future studies incorporating longer observation periods, biofilm-mediated caries models, and pulpal response assessments are needed to further clarify the clinical implications of temporary cement selection in deep caries management.^39^ The present in vitro pH-cycling model does not fully reproduce the biological complexity of deep caries lesions, including pulpal responses, dentinal fluid movement, biofilm activity, and long-term intraoral conditions. Therefore, direct clinical extrapolation should be interpreted cautiously. Furthermore, SS-OCT attenuation coefficients represent indirect optical measurements associated with changes in light scattering behaviour and should not be interpreted as direct evidence of dentin remineralization. In addition, the cement removal procedure performed prior to analysis may have partially influenced superficial deposits and surface morphology observed in SEM/EDS images. Nevertheless, the combined evaluation using SS-OCT, TMR, SEM/EDS, and ion release analysis provided complementary insights into the protective effects of ion-releasing cements on demineralized dentin under the present experimental conditions.

## Conclusion

Ion-releasing cements demonstrated protective effects on demineralized dentin under the present in vitro pH-cycling conditions. The findings suggest reduced lesion progression and mineral deposition-related changes, particularly in the S-PRG filler–containing temporary cement group. Although further studies are required to evaluate long-term biological and mechanical effects under clinically relevant conditions, these results support the potential role of ion-releasing temporary materials in preserving demineralized dentin when definitive restoration must be delayed.

## Funding

This research did not receive any specific grant from funding agencies in the public, commercial, or not-for-profit sectors.

## Author contributions

All authors contributed to the study conception and design. Material preparation, data collection, and analysis were performed by Zijuan D, Tomoko T, Noriko H, and Yasushi S. The first draft of the manuscript was written by Zijuan D and Tomoko T, and all authors commented on previous versions of the manuscript. All authors read and approved the final manuscript.

## Conflict of interest

The authors declare that they have no known competing financial interests or personal relationships that could have influenced the work reported in this article.

## Appendix Supplementary materials

Image, application 1

## Footnotes

Supplementary material associated with this article can be found in the online version at doi:10.1016/j.identj.2026.109753.
