# Supplementary material for: Remineralization-Related Effects of Multi-Ion Cements on Demineralized Dentin
Source: Int Dent J. 2026 Jul 16;76(5):109753. doi: 10.1016/j.identj.2026.109753 (PMC13400383; doi:10.1016/j.identj.2026.109753)
Supplement: Supplementary file 1 [file mmc1.docx]

**Table S1. Ion release profiles of tested materials determined by ICP-AES and a fluoride-selective electrode.**

| mg/L | B | P | Al | Ca | Zn | Sr | Fluoride |
| --- | --- | --- | --- | --- | --- | --- | --- |
| CR (Caredyne Restore) | 6.55 | 12.20 | 705.36 | 382.68 | 1937.42 | 738.46 | 13.5 |
| FU (Fuji IX) | 0 | 13.44 | 639.52 | 0 | 0 | 1758.98 | 8.86 |
| HY (HY Bond Temporary Cement Hard) | 0 | 17.14 | 0 | 0 | 13876.1 | 0.058 | 139.17 |
| IP (IP Temp Cement) | 104.43 | 15.20 | 22.42 | 0 | 12900 | 306.04 | 33.08 |

Ion release was analyzed using inductively coupled plasma atomic emission spectroscopy (ICP-AES, ICPS-8100, Shimadzu Corporation, Japan) and a fluoride-selective electrode (HORIBA, Kyoto, Japan). Reported concentrations were corrected for the 50-fold dilution and expressed as concentrations in the undiluted (100%) eluates.

Because Zn concentrations were extremely high even after dilution, the absolute values should be interpreted with caution.
